# Bone Mineral Density in Adults With Cerebral Palsy

**DOI:** 10.3389/fneur.2021.733322

**Published:** 2021-09-10

**Authors:** Jun Hee Won, Se Hee Jung

**Affiliations:** ^1^Department of Rehabilitation Medicine, Seoul National University College of Medicine, Seoul, South Korea; ^2^Department of Rehabilitation Medicine, Seoul National University Boramae Medical Center, Seoul, South Korea

**Keywords:** cerebral palsy, osteoporosis, bone density, adult, cerebral palsy - complications

## Abstract

Low bone mineral density (BMD) is an emerging health issue in adults with cerebral palsy (CP). This cross-sectional study aimed to describe the characteristics of BMD in adults with CP, and to elucidate the risk factors for low BMD in this population. People aged ≥20 years and diagnosed with CP were recruited from February 2014 to November 2014. We assessed BMD using dual-energy X-ray absorptiometry (DXA) for the lumbar spine, femoral neck, and total femur. Moreover, the body composition was assessed using DXA. We included a total of 87 adults with CP (mean age 42.01 years; 52 men). The prevalence of low BMD was 25.3%. Male sex and age were associated with lower BMD. BMD was significantly lower in the non-ambulatory group than that in the ambulatory group for both lumbar spine and femoral neck. The total fat mass demonstrated a positive correlation with the *Z*-score and BMD for the femur neck and total femur. Body mass index (BMI) and total fat mass were positively correlated with BMD in the lumbar spine, femoral neck, and total femur. However, the Gross Motor Function Classification Scale levels were negatively correlated with BMD at the aforementioned three sites. In conclusion, adults with CP revealed decreased BMD, which was associated with male sex, age, decreased gross motor function, loss of ambulatory function, low BMI, decreased total fat mass, and decreased total fat-free mass.

## Introduction

Low bone mineral density (BMD) is a common health issue in cerebral palsy (CP) (1, 2). Children with CP have lower BMD than their non-disabled counterparts (2). Several causes, including decreased ambulation, insufficient vitamin D or calcium, or anticonvulsants, supposedly induce low BMD in children and adolescents with CP (3). In addition, individuals with CP demonstrate a higher prevalence of fracture (4) and increased risk of fracture with minor stress (5). Fracture impedes ambulation and affects their quality of life (6). This necessitates BMD assessment in adults with CP.

There is little evidence for BMD and its associated factors in adults with CP. Adults with CP are more likely to experience reduced physical activities throughout their lives. Therefore, decreased mechanical force on the bone could decrease the anabolic influence on bone metabolism, besides causing low BMD (7). Several studies have reported on low BMD in young adults with CP. Young adults with CP demonstrate relatively low bone mass in the lumbar spine, hip, and femur (7, 8). In addition, BMD of young adults with CP is associated with ambulatory status (9), types of CP (7), and the Gross Motor Function Classification Scale (GMFCS) scores (10). However, these studies were either confined to non-ambulatory participants (8) or young populations (9–11). Despite previous reports on correlation between the body mass index (BMI) and higher BMD (9–11), researchers have not investigated the association between body composition and BMD in adults with CP.

Therefore, we aimed to investigate BMD in adults with CP and to investigate the factors related to BMD, including body composition variables.

## Methods

### Study Design and Population

A total of 243 adults with CP were identified from outpatient rehabilitation clinics of the participating hospitals and were referred to our clinic. All participants were aged ≥20 years. The exclusion criteria were as follows: (i) inability to understand or respond to our written questionnaire even with the aid of an interviewer, (ii) failure to complete dual-energy X-ray absorptiometry (DXA), or (iii) participation withdrawal prior to data collection. Data were collected between February 1, 2014, and November 31, 2014. All procedures were approved by the institutional review boards of the participating institutions and were in compliance with the guidelines for good clinical practice. All procedures were in accordance with the ethical standards of the institutional and national research committees, and with the tenets of the 1964 Declaration of Helsinki.

### Assessment Procedure

We requested the participants to complete questionnaires on their demographics, physical function, and socioeconomic background. A registered nurse measured basic body anthropometries, such as height, weight, and waist circumference. A physiatrist with 15 years of clinical experience in CP (S.H.J.) conducted structured interviews. Clinical variables, such as the GMFCS level and manual muscle strength, were assessed by a physiatrist.

The body composition was assessed using a single DXA machine (GE Lunar Prodigy, Bedford, MA, USA). The lumbar spine and hip joints were imaged and analyzed using the software of the manufacturer. BMD was measured in grams per square centimeter and was represented using *Z*-scores and *T*-scores. The *Z*-score refers to the number of standard deviations, compared with age- and sex-matched individuals. The *T*-score refers to those compared with sex-matched individuals aged 30 years. If the bone mineral density of an individual is lower than the average of the reference group, the *T*-score, and *Z*-score are smaller than 0. A *Z*-score below −2.0 at any of the lumbar spine (LS), total femur (TF), and femur neck (FN) regions was classified “below the expected range for age” (low BMD) (12). Based on the *T*-score, we classified BMD into the following three categories according to the World Health Organization criteria: ≥–1.0, normal; > –2.5 and < –1.0, osteopenia; and ≤ –2.5, osteoporosis. Clinicians recommend treatment for subjects classified as having low BMD or osteoporosis to reduce the risk of fracture and morbidity. *Z*-scores are recommended for adolescents and young adults (12), and most studies have previously reported on *Z*-scores for BMD of adults with CP (7, 9, 10). Therefore, we reported on *Z*-scores as the major outcome. However, we also reported on the *T*-score because ~ half of the participants were older than 40 years. The total fat mass and total fat-free mass were also assessed using DXA. The availability and cost-effectiveness of analyzing body composition by DXA scans have been reported in previous studies (13, 14).

### Statistical Analyses

To identify the difference in *Z*-scores and the proportion of low BMD between groups, we performed a univariate analysis for nominal variables, including sex, ambulatory function, and involvement site. We compared BMD across the GMFCS levels using an analysis of variance. We analyzed the correlation between *Z*-scores and BMD for each site and other clinical information using Pearson correlation analysis. A stepwise selection of variables was conducted using multiple linear regression analysis. The independent variables were those that significantly correlated with BMD. In contrast, BMD of the LS, TF, and FN regions were the dependent variables. All statistical analyses were conducted using SPSS ver. 25.0 (SPSS Inc., Chicago, IL, USA).

## Results

### Study Population

A total of 87 adults with CP were included in this study. The mean age was 42.01 ± 8.29 years (age range, 23–68 years). Of these participants, 49.4% were 40 years old or younger, 38.0% were aged between 41 and 50 years. There were 52 men (mean age: 42.63 ± 9.33 years) and 35 women (mean age: 41.09 ± 6.46 years). The spastic type was the most common type of CP (31 subjects; 35.6%), followed by the mixed type (28 subjects; 32.2%). Table 1 summarizes the detailed characteristics of the study population.

**Table 1:** Characteristics of the study population.

		**Total (*N* = 87)**
Age	≤ 40	43 (49.4%)
	>40, ≤ 50	33 (38.0%)
	>50	11 (12.6%)
Sex	Male	52 (59.8%)
	Female	35 (40.2%)
BMI	<20	24 (27.6%)
	≥20, <25	40 (46.0%)
	≥25	23 (26.4%)
Type	Spastic	31 (35.6%)
	Mixed	28 (32.2%)
	Dystonic	13 (14.9%)
	Dyskinetic	8 (9.2%)
	Ataxic	1 (1.1%)
	Others	6 (6.9%)
Involvement	Bilateral	64 (73.6%)
	Unilateral	20 (23.0%)
	Not known	3 (3.4%)
GMFCS	I	10 (11.5%)
	II	21 (24.1%)
	III	4 (4.6%)
	IV	46 (52.9%)
	V	6 (6.95%)
Ambulation	Ambulatory	35 (40.2%)
	Non-ambulatory	52 (59.8%)

*BMI, body mass index; GMFCS, Gross Motor Function Classification System*.

### BMD of the Study Population

**Figure 1:**
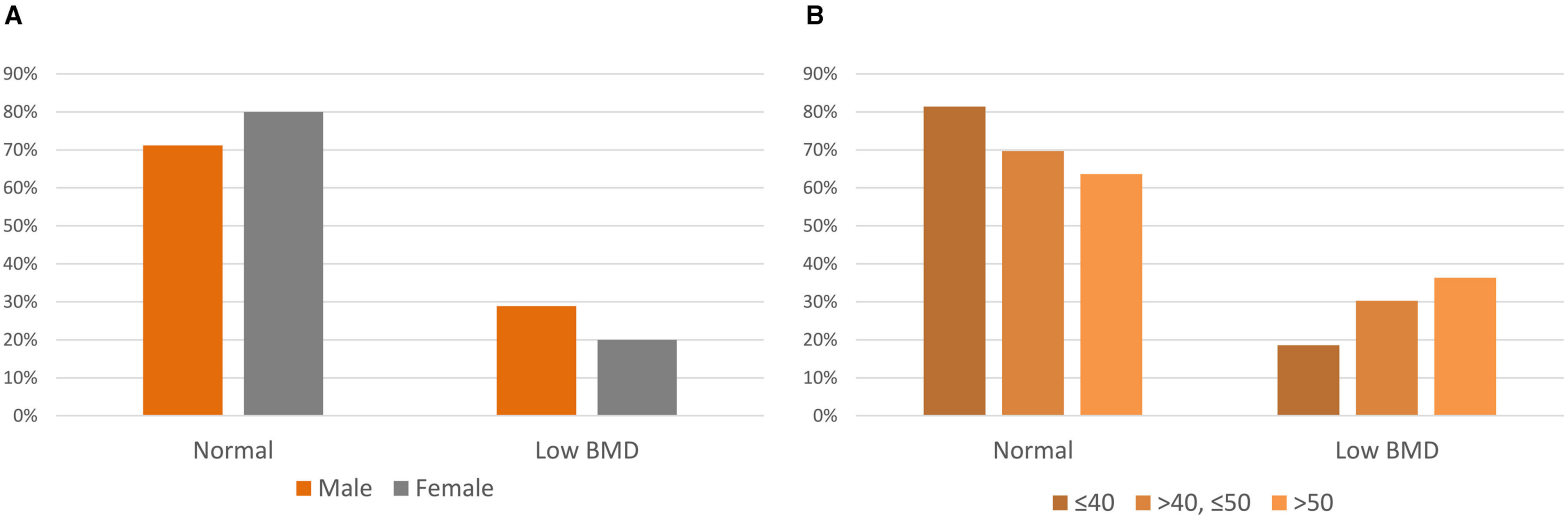
*Z*-scores of adults with cerebral palsy. **(A)***Z*-scores by sex. **(B)***Z*-scores by the age group.

The average *Z*-scores were −0.64 ± 1.49, −0.76 ± 1.13, and −0.45 ± 1.21 for the LS, TF, and FN regions, respectively. Twenty-two subjects (25.3%) revealed *Z*-scores below −2.0 at any of the LS, TF, and FN regions. A *Z*-score below −2.0 was observed in 29% of the men and 20% of women (Figure 1A). By age group, 19% of subjects were 40 years or younger, 30% were 50 years or younger, and 36% were older than 50 years and had a *Z*-score below −2.0 (Figure 1B).

**Figure 2:**
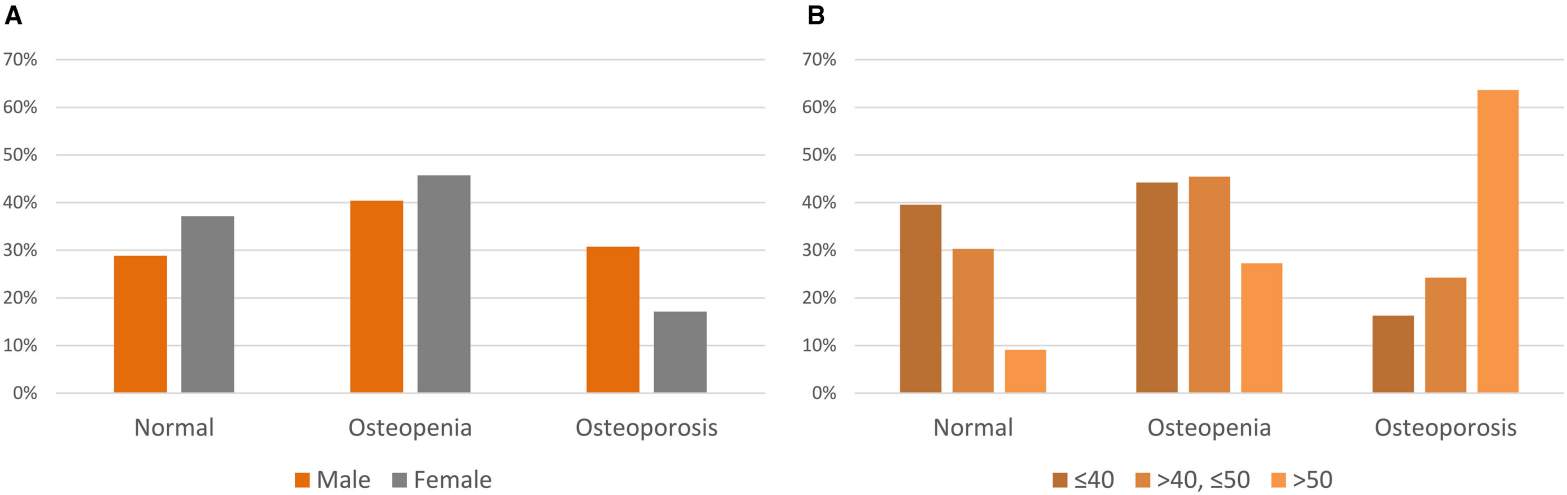
*T*-scores of adults with cerebral palsy. **(A)***T*-scores by sex. **(B)***T*-scores by age group.

The average *T*-scores were −0.91 ± 1.74, −1.03 ± 1.25, and −1.17 ± 1.37 for the LS, TF, and FN, respectively. Thirty-four (39.1%) and 21 (24.1%) subjects were classified as having osteopenia and osteoporosis, respectively. Osteopenia and osteoporosis were prevalent in 40.0 and 30.8% men and 45.7, and 17.1% women, respectively (Figure 2A). Osteopenia and osteoporosis accounted for 44 and 16% of the subjects aged ≤40 years, 45 and 24% in subjects aged ≤50 years, and 27 and 64% in subjects aged >50 years, respectively (Figure 2B).

### Factors Associated With BMD in Adults With CP

The mean LS *Z*-score was significantly lower in men than that in women (*p* < 0.001). However, *Z*-scores in the FN and TF regions did not display significant differences by sex. There was no significant difference in *Z*-scores between bilateral CP and unilateral CP. Subjects with bilateral involvement had a significantly higher prevalence of *Z*-score below −2.0 than those with unilateral involvement. The *Z*-score was insignificantly different between those with non-ambulatory and ambulatory CP. Non-ambulatory subjects had a significantly higher prevalence of *Z*-score below −2.0 in the LS and FN regions. The mean *Z*-score was significantly different between subjects with GMFCS level I and GMFCS level V in the FN (*p* = 0.03) and TF regions (*p* = 0.005). Table 2 summarizes the *Z*-scores and low BMD.

**Table 2:** *Z*-scores and “low BMD” group for sex, involvement site, and ambulatory status.

	***N***	**Lumbar spine**		**Femoral neck**		**Total femur**	
		**Mean ± SD**	***Z*-score below −2.0**	**Mean ± SD**	***Z*-score below −2.0**	**Mean ± SD**	***Z*-score below −2.0**
**Total**	87	−0.64 ± 1.49	13 (15.0%)	−0.78 ± 1.21	12 (13.8%)	−0.76 ± 1.13	11 (12.6%)
**Sex**
Male	52	−1.09 ± 1.35^*^	12 (23.1%)^*^	−0.77 ± 1.22	7 (13.5%)	−0.56 ± 1.08	5 (9.6%)
Female	35	0.04 ± 1.45^*^	1 (2.9%)^*^	−0.79 ± 1.21	5 (14.3%)	−1.04 ± 1.17	6 (17.1%)
**Involvement site**
Bilateral	64	−0.67 ± 1.63	11 (17.2%)	−0.71 ± 1.30	11 (17.2%)^*^	−0.69 ± 1.21	10 (15.6%)
Unilateral	20	−0.48 ± 0.98	1 (5.0%)	−0.99 ± 0.81	0 (0%)^*^	−0.98 ± 0.86	1 (5.0%)
**Ambulatory status**
Ambulatory	35	−0.39 ± 1.32	2 (5.7%)^*^	−0.46 ± 0.95	1 (2.9%)^*^	−0.57 ± 0.89	2 (5.7%)
Non–ambulatory	52	−0.82 ± 1.64	11 (21.1%)^*^	−1.00 ± 1.33	11 (21.2%)^*^	−0.88 ± 1.26	9 (17.3%)
**GMFCS**
I	10	−0.15 ± 0.58	1 (10.0%)	−0.04 ± 0.35	0 (0.0%)	−0.06 ± 0.28	0 (0.0%)
II	21	−0.41 ± 0.24	1 (4.8%)	−0.77 ± 0.17	1 (4.8%)	−0.90 ± 0.18	2 (9.5%)
III	4	−0.91 ± 0.42	0 (0.0%)	−0.08 ± 0.46	0 (0.0%)	−0.08 ± 0.19	0 (0.0%)
IV	46	−0.72 ± 0.25	9 (19.6%)	−0.83 ± 0.18	8 (17.4%)	−0.68 ± 0.18	6 (13.0%)
V	6	−1.89 ± 0.33	2 (33.3%)	−1.70 ± 0.86^*^	3 (50.0%)	–2.40 ± 0.46^*^	0.5 (50.0%)

*SD, standard deviation; GMFCS, Gross Motor Function Classification System*.

* *p-value < 0.05*.

Table 3 outlines the factors correlated with BMD and *Z*-score of each region. Subjects with a higher body fat percentage displayed higher *Z*-scores in the LS region. In contrast, subjects with higher total fat-free mass and those with a lower GMFCS demonstrated higher *Z*-scores in the FN region. Moreover, those with higher BMI, higher total fat-free mass, and lower GMFCS displayed higher *Z*-scores in the TF region. The waist circumference did not display any correlation with the *Z*-scores in the aforementioned three regions.

**Table 3:** Pearson correlation and *p*-values for *Z*-scores, and bone mineral density.

	**Lumbar spine**		**Femur neck**		**Total femur**	
	***Z*-score**	**BMD**	***Z*-score**	**BMD**	***Z*-score**	**BMD**
Age	0.046 (0.681)	−0.173 (0.121)	−0.047 (0.67)	−0.283 (0.009)^*^	−0.021 (0.846)	−0.229 (0.035)^*^
BMI	0.119 (0.289)	0.447 (<0.001)^*^	0.083 (0.453)	0.301 (0.005)^*^	0.218 (0.047)^*^	0.439 (<0.001)^*^
WC	−0.045 (0.707)	0.099 (0.410)	−0.028 (0.811)	0.049 (0.676)	0.085 (0.470)	0.159 (0.172)
Total fat-free mass	−0.185 (0.115)	0.028 (0.814)	0.333 (0.003)^*^	0.332 (0.003)^*^	0.418 (<0.001)^*^	0.312 (0.006)^*^
Total fat mass	0.145 (0.219)	0.4770 (<0.001)^*^	0.05 (0.665)	0.330 (0.003)^*^	0.109 (0.346)	0.429 (<0.001)^*^
Percent body fat	0.337 (0.004)^*^	0.558 (<0.001)^*^	0.048 (0.678)	0.306 (0.007)^*^	0.067 (0.566)	0.401 (<0.001)^*^

*BMD, bone mineral density; BMI, body mass index; WC, waist circumference*.

* *p-value < 0.05*.

Multiple linear regression using stepwise selection revealed total fat-free mass as a significant factor associated with BMD in the TF and FN regions. In addition, sex was significantly associated with BMD in the LS and TF regions (Table 4).

**Table 4:** Multiple linear regression analysis for *Z*-scores in subjects with cerebral palsy.

	**Lumbar spine**			**Femur neck**			**Total femur**		
	**Coefficient**	**SE**	***p*-value**	**Coefficient**	**SE**	***p*-value**	**Coefficient**	**SE**	***p*-value**
Intercept	−1.069	1.757	0.545	−3.621	1.343	0.009	−2.841	1.281	0.03
Age	0.025	0.021	0.234	−0.007	0.016	0.667	0	0.015	0.993
Sex^a^	−1.315	0.546	0.019	−0.974	0.42	0.023	−0.178	0.401	0.658
Involvement site^b^	0.131	0.419	0.755	−0.242	0.319	0.45	−0.073	0.304	0.812
GMFCS	−0.38	0.393	0.337	0.157	0.296	0.599	−0.236	0.283	0.407
Ambulatory status^c^	0.873	0.924	0.349	−0.495	0.706	0.485	0.402	0.673	0.553
Total fat mass	0.015	0.023	0.511	−0.025	0.017	0.145	−0.001	0.016	0.936
Total fat-free mass	0.016	0.037	0.676	0.099	0.028	<0.001	0.068	0.027	0.012

*SE, standard error; GMFCS, Gross Motor Function Classification System*.

a *Nominal variable (male = 1, female = 0)*.

b *Nominal variable (bilateral = 0, unilateral = 1)*.

c *Nominal variable (ambulatory = 0, non-ambulatory = 1)*.

^*^p-value < 0.05.

## Discussion

BMD by *Z*- and *T*-scores of adults with CP was lower than that of the age- and sex-matched general population. Moreover, ~25% of the participants had a *Z*-score below normal in either the LS, FN, or TF regions. Based on the *T*-scores, 24% of the participants and 64% of those older than 50 years were classified as having osteoporosis. Men, non-ambulators, and bilateral CP were more likely to have lower *Z*-scores. In addition, adults with lower BMI, lower total fat-free mass, lower percent body fat, and higher GMFCS levels had a lower *Z*-score.

This is the first study to investigate the association between BMD and body composition, including BMI, total fat mass, and fat-free mass, in adults with CP. BMI was identified as a significant factor for BMD in the entire region and for the *Z*-scores in the TF region. Total fat mass was another significant factor for BMD in the LS, FN, and TF regions. BMI was previously reported as an important determinant of BMD in the general population (15), and in subjects with CP (9–11). In the general population, BMD increases with BMI in weight-bearing bones (16). A study reported on a correlation between lower triceps skin-fold measurement, and a lower BMD (17). The triceps skin-fold measurement is an indirect measurement of body fat mass. Thus, our results provided more direct evidence for the association between total fat mass and BMD. Another possible explanation is nutritional status. Individuals with higher total fat mass and BMI supposedly have better nutritional status than those with lower total fat mass or BMI (18). Total fat-free mass was correlated with both *Z*-scores and BMD of the FN and TF regions. Moreover, it was significantly associated with higher BMD in the multiple linear regression analysis of the FN and TF regions. Total fat-free mass could also affect BMD by increasing weight loading. Moreover, it is considered a predictor of muscle strength (19). The force exerted against the bone could affect bone mass and BMD. In addition, a larger total fat-free mass could be interpreted as being of greater physical activity level. A meta-analysis reported that both fat mass and fat-free mass were positively correlated with BMD in the general population (18). Our findings provided similar evidence in individuals with CP. Therefore, BMD maintenance might necessitate maintaining proper fat mass and fat-free mass through nutrition support and physical therapy.

The non-ambulatory subjects displayed a significantly higher prevalence of low *Z*-scores in the LS and FN regions. Bilateral CP revealed a significantly higher prevalence of low *Z*-scores than unilateral CP. In addition, individuals with higher GMFCS levels demonstrated lower *Z*-scores in the FN and TF regions. Ambulatory status is one of the best indicators of weight-bearing status. Several studies reported that BMD of the weight-bearing bone in the CP population is associated with their ambulatory status and GMFCS level. Fowler et al. mentioned that *Z*-scores of the LS, TF, and FN regions were significantly lower in non-ambulatory adults with CP (10). Yoon et al. reported that ambulatory subjects had higher *T*-scores in the hip region, but not in the LS region (11). Our data are consistent with those of previous studies. Higher GMFCS levels and non-ambulatory status were not only associated with lower BMD but also with a higher risk of fractures in children with CP (20). This necessitates regularly assessing BMD in non-ambulatory adults with CP to prevent fractures.

Previous reports on the relationship between age and BMD in adults with CP are inconsistent. According to Fowler et al., *Z*-scores of the LS and hip increased with age in adults with CP (10). However, two other studies reported on no association between BMD and age (8, 11). Our findings shed light on a negative association between BMD and age in adults with CP. However, this correlation is relatively small, thus warranting a larger cohort to elucidate these relationships.

The mean *Z*-score was −0.64 and ~25% of adults with CP had *Z*-scores below the expected range for age and sex. In previous studies, the prevalence of low *Z*-scores was reportedly >58% (8) and 50% (9) in the LS region. Fowler et al. (10) mentioned that the average *Z*-scores were −1.40, −1.36, and −1.02 for the LS, TF, and FN regions, respectively. Other studies reported average *Z*-scores of −2.37 (8) and −1.69 (9) in the LS region. The average *Z*-score in this study was higher than that in other studies. Our findings also revealed a higher average *T*-score. Yoon et al. (11) reported an average *T*-score of −1.08 and −1.50 for the LS and TF regions, respectively, by investigating 35 subjects with CP (mean age, 35.18 ± 1.87 years). This difference might account for the differences in the study cohort. Considering the enrollment of adults in nationwide organizations for disabled people, the study population might have had a higher level of physical activity, and better medical status than those in other studies.

This study had several limitations. First, the study population did not represent the entire CP population in South Korea. Second, considering the cross-sectional design, we could not investigate the causal relationship between the clinical characteristics and low BMD. Third, we did not investigate the longitudinal course of BMD and events, including falls and fractures after recruitment. Lastly, we could not collect medication histories from the participants, such as anticonvulsive medications. Therefore, we could not establish a direct association between each factor and the risk of fracture.

In conclusion, adults with CP had a lower BMD than the sex- and age-matched general population. Those with CP and lower BMI, lower total fat-free mass, and lower total fat mass were more likely to have lower *Z*-scores. Decreased BMD in adults with CP was associated with male sex, age, decreased gross motor function, and the loss of ambulatory function.

## Data Availability Statement

The datasets presented in this article are not readily available because the datasets generated during and/or analysed during the current study are not expected to be made available publicly, as consent was not obtained to publish the anonymised data. Requests to access the datasets should be directed to Se Hee Jung, ideale1@snu.ac.kr.

## Ethics Statement

The studies involving human participants were reviewed and approved by Seoul National University Boramae Medical Center Institutional Review Board. All subjects provided their written informed consent to participate in this study.

## Author Contributions

SJ conceived and designed the study, collected the data, interpreted the data, and revised the manuscript for intellectual content. JW performed the analysis and drafted the manuscript. Both authors contributed to the article and approved the submitted version.

## Funding

This work was supported by a focused clinical research grant-in-aid from the Seoul Metropolitan Government Seoul National University (SMG-SNU) Boramae Medical Center (03-2021-0014).

## Conflict of Interest

The authors declare that the research was conducted in the absence of any commercial or financial relationships that could be construed as a potential conflict of interest.

## Publisher's Note

All claims expressed in this article are solely those of the authors and do not necessarily represent those of their affiliated organizations, or those of the publisher, the editors and the reviewers. Any product that may be evaluated in this article, or claim that may be made by its manufacturer, is not guaranteed or endorsed by the publisher.
